# Debate: Where to next for universal school‐based mental health interventions? The value of student voices in informing the design and implementation of universal school‐based mental health interventions

**DOI:** 10.1111/camh.12750

**Published:** 2025-01-13

**Authors:** Emma Carter

**Affiliations:** ^1^ Assessment and Evaluation Research Centre, Faculty of Education University of Melbourne Melbourne Vic. Australia

**Keywords:** Mental health, school, intervention, methodology, education

## Abstract

Universal school‐based mental health interventions present a promising approach to addressing youth mental health challenges; however, evidence suggests their effectiveness is often limited and unsustained. One contributing factor to this issue is the frequent superficial involvement of students in the design, implementation and evaluation of initiatives. In response, this paper advocates for the urgent prioritisation of student voice within these programmes, using in‐depth and purposive qualitative approaches that empower youth to actively co‐create and shape interventions. Research highlights that incorporating student voice can yield vital insights into what is effective and ineffective in programmes, particularly for marginalised groups. Moreover, engaging students in co‐designing methodologies that help amplify their voices ensures research processes are better aligned with their needs and preferences, fostering greater ownership and improved outcomes. This shift, which repositions students as active collaborators rather than passive recipients, has the potential to enhance both the effectiveness and equity of initiatives.

1

Universal school‐based mental health interventions, designed to be accessible to all students regardless of their current mental health status, have emerged as a promising strategy in addressing youth mental disorders. By providing early preventive measures, these interventions have the potential to not only help mitigate the onset of mental health issues but also work to reduce stigma associated with school students seeking mental health support through separate sessions. A growing body of research from varied international contexts indicates their effectiveness in alleviating symptoms such as depression and anxiety among youth; however, the extent of this is often modest and short‐lived (Zbukvic et al., 2024). Other expressed concerns relating to universal school‐based mental health interventions include their potential to exacerbate existing symptoms among students, such as inattention/hyperactivity and emotional problems, following exposure (Foulkes & Stapley, 2022; Kuyken et al., 2022). A further critical issue of relevance to the current paper is the lack of student engagement in programmes, which likely impacts effectiveness (Montero‐Marin et al., 2022).

One primary reason attributed to observed low levels of student engagement is the absence or superficial nature of their involvement in developing, implementing and evaluating universal school‐based mental health initiatives (Carlson et al., 2021; Montero‐Marin et al., 2022). Despite attempts to solicit student perspectives on these areas, for example, via survey responses, they tend to lack depth, focusing primarily on enhancing the experience of taking sessions rather than providing substantive contributions to the programme's design and improvement (Foulkes & Stapley, 2022). In reference to Hart's (1992) seminal ‘Ladder of Children's Participation’, many initiatives remain trapped in ‘tokenism’ or, at best, achieve a ‘consultative’ level of involvement. Rarely do these efforts facilitate meaningful participation where students feel their voices are genuinely heard and valued.

Related to this issue, the existing evidence‐base reveals limited attention to how methodological design can be most effectively used to inform and improve interventions via the use of student voice (Bailey et al., 2023; Foulkes & Stapley, 2022). For instance, quantitative findings in an existing mixed‐method study highlighted important issues, such as greater engagement from students with mental health challenges compared to those without and perceived stronger relevance of programmes by boys relative to girls. However, there was no qualitative follow‐up to explore these findings with relevant student subgroups in greater detail (Bailey et al., 2023). This underscores the need for more in‐depth and purposive qualitative research which promotes student voice, and a stronger awareness of how methodological design can help to maximise research findings and impacts (Bailey et al., 2023; Halliday, Kern, Garrett, & Turnbull, 2019).

Student voice in its most genuine form involves enabling youth to recognise and analyse issues related to their development which they regard as meaningful and having their voices inform action (Fielding & Bragg, 2003, cited in Cook‐Sather, 2020, p. 182). It can be enacted in various ways, such as via students participating in decision‐making processes, giving feedback and being involved in programme design. It can also occur via a number of qualitative research methods including interviews, focus group discussions, written reflections and open‐ended surveys. Student voice has the potential to open‐up opportunities for youth from marginalised and minority groups to play key roles in changes impacting their development and well‐being (Cook‐Sather, 2020). In the context of mental health programmes, integrating student voice means actively listening to their experiences, understanding their needs and tailoring interventions accordingly. When students have a say in shaping the programmes that directly affect them, they are more likely to feel valued and engaged, leading to better outcomes (Foulkes & Stapley, 2022).

While still emerging, the literature highlights the critical role that student voice can play in universal school‐based mental health programme design, implementation and evaluation. For example, a recent ethnographic study conducted in Uganda utilising in‐depth interviews with students revealed that complementing universal interventions with peer group opportunities may help reduce the fear of discussing difficulties related to mental health and help guide younger students. Within this study, students expressed concerns about opening‐up to authority figures such as teachers, who commonly facilitate programme sessions, suggesting that peer facilitators could create a more comfortable environment whereby students help and support one another with their problems (Carlson et al., 2021). Complementing this research are practical guidelines that support the use of student voice. For example, in drawing on a range of evidence examples, the Orygen toolkit on including student voice in school‐based mental health programmes outlines numerous benefits that can arise when programmes are co‐designed by youth including increased participation, improved satisfaction in programmes, decreased dropout as well as the development of stronger relationships between students and facilitators (See Anderson, Cooke, & Zbukvic, 2020, for review).

Concerning methodological design, researchers could also benefit from paying closer attention to how qualitative techniques using student voice can be best used as either a stand‐alone method within research focused on understanding and improving universal school‐based mental health interventions or in combination with quantitative approaches in mixed‐method studies (Bailey et al., 2023). This involves giving greater consideration to diverse student populations and relatedly, the dynamics of group compositions within research to gain deeper insights into programme design and implementation challenges. For example, targeted focus groups could provide critical insights by centring the perspectives of specific populations identified in prior studies as having unique concerns, such as girls, LGBT+ students or students with and without reported mental health challenges (Bailey et al., 2023; Foulkes & Stapley, 2022). Such tailored groups could foster safer environments, enabling participants to discuss sensitive topics more freely compared to mixed groups, where they may feel more exposed. Furthermore, the potential impact of power dynamics when engaging with vulnerable youth through qualitative research techniques encouraging student voice should not be underestimated nor overlooked. Alternative strategies for building trust and rapport through the feedback process, such as through enabling more private individual written reflections on experiences or employing student‐derived visual stimuli to facilitate sensitive discussions, could help promote a greater sense of student ownership over universal school‐based mental health interventions and increased openness when expressing perspectives and recommendations. Importantly, researchers should actively involve students in determining the most appropriate formats and settings for engagement to ensure the research process aligns with their preferences and needs (Anderson et al., 2020).

In conclusion, the journey towards effective universal school‐based mental health interventions requires a shift in perspective. Instead of viewing students merely as recipients of support, we must recognise them as active participants in the development, implementation and evaluation of programmes that affect their lives. By fostering environments and processes where their voices are heard and valued, we can create more impactful interventions that not only address immediate mental health concerns but also empower students to take charge of their well‐being. This shift is crucial for ensuring that universal approaches to mental health in schools are not only effective but responsive to the needs of all students.

## 2 Conflict of interest statement

The author has declared that they have no competing or potential conflicts of interest.

## 3 Ethical information

No ethical approval was required for this article.

4

## Data Availability

Data sharing is not applicable to this article as no new data were created or analyzed in this study.
